# Pathotypes and Phenotypic Resistance to Antimicrobials of *Escherichia coli* Isolates from One-Day-Old Chickens

**DOI:** 10.3390/pathogens12111330

**Published:** 2023-11-08

**Authors:** Katerina Nedbalcova, Jaroslav Bzdil, Aneta Papouskova, Monika Zouharova, Katarina Matiaskova, Kamil Stastny, Vladimir Sladecek, David Senk, Matej Petr, Petr Stolar

**Affiliations:** 1 Veterinary Research Institute, Hudcova 296/70, 621 00 Brno, Czech Republic; monika.zouharova@vri.cz (M.Z.); katarina.matiaskova@vri.cz (K.M.); kamil.stastny@vri.cz (K.S.); 2 Ptacy S.R.O., Valasska Bystrice 194, 756 27 Valašská Bystřice, Czech Republic; vetmed@seznam.cz (J.B.); sladecek.vladimir@gmail.com (V.S.); dsenk@post.cz (D.S.); ptacy@ptacy.cz (M.P.); drstolar@seznam.cz (P.S.); 3 Institute of Infectious Diseases and Microbiology, Faculty of Veterinary Medicine, University of Veterinary Sciences, Palackeho 1–3, 612 42 Brno, Czech Republic; papouskovaa@vfu.cz

**Keywords:** poultry, pathogenicity, virulence, avian pathogenic *E. coli*, multidrug resistance, prevention

## Abstract

The aim of this work was to describe the pathotypes of *Escherichia coli* strains isolated from one-day-old chickens, as well as the occurrence of resistance and multidrug resistance (MDR) in these strains. A total of 429 mixed swabs from 4290 one-day-old chicks were examined between August 2021 and July 2023 (24 months) during routine point-of-destination inspections at 12 poultry farms in the Czech Republic. All samples were processed via cultivation methods using meat-peptone blood agar and Mc Conkey agar under aerobic conditions at 37 ± 1 °C for 18–24 h. The identification of the strains was performed using MALDI-TOF mass spectrometry. All confirmed strains of *E. coli* were screened via single or multiplex PCRs for the presence of genes encoding the virulence-associated factors *iroN*, *cvaC*, *iss*, *felA*, *iutA*, *frz* and *tsh.* Antimicrobial susceptibility tests were performed using the minimal inhibitory concentration (MIC) method, focusing on ampicillin, cefotaxime, tetracycline, doxycycline, enrofloxacin, florfenicol, amoxicillin with clavulanic acid and trimethoprim with sulfamethoxazole. A total of 321 *E. coli* strains (prevalence of 74.8%) were isolated, and 300 isolates were defined as avian pathogenic strains of *E. coli* (APEC) via multiplex PCR. Based on the defined virulence genes, the isolates were classified into 31 pathotypes. A total of 15.9% of the tested isolates were susceptible to all the tested antimicrobials. On the other hand, 20.5% of the isolates were identified as multidrug-resistant (8.7% of isolates were resistant to three antimicrobials, 7.3% to four antimicrobials, 3.6% to five antimicrobials and 0.9% to six antimicrobials). Monitoring pathogenic strains of *E. coli* in different animals and in the environment makes it possible to understand their spread in animal and human populations and, at the same time, reveal the sources of virulence and resistance genes.

## 1. Introduction

Colibacillosis, caused by avian pathogenic strains of *Escherichia coli* (APEC), is considered the most common bacterial infection of poultry with the most serious economic impact on poultry production. It is a complex disease of all ages and production categories with a septicemic course and significant losses. In broiler breeders, yolk sac infection and early mortality are the most common manifestations, followed by air sac inflammation and polyserositis from about 2 weeks of age. The severity of the course depends, to varying degrees, on the virulence of the causative agent and a number of predisposing factors (zoohygiene, stress, immunosuppression, etc.) [1]. In addition to the negative health and economic impacts, APEC isolates are also considered one of the main sources of spread of antimicrobial resistance to other bacterial species, mainly through their plasmids and the exchange of other genetic material. The literature reports that up to 92% of APEC isolates are resistant to three or more antimicrobials, despite strict measures regarding antibiotic use in the poultry industry [2].

Recently, the virulence of APEC isolates has been associated with a number of genetically encoded factors. These virulence genes may play roles in various aspects of the extraintestinal pathogenesis of APEC, and their functions can be categorized as adhesion, iron acquisition, hemolysis, protection from bactericidal host factors and toxin production. The diagnostic PCR methods are based on the detection of these genes to determine whether clinical *E. coli* isolates can be classified as APEC. However, clinical isolates are extremely variable and do not always show signs of typical APEC [3,4,5,6,7]. Even among authors of published papers, there is still no consensus on which genes are ideal markers of virulence [8]. For the purposes of our study, to evaluate the variability in potential virulence genotypes in day-old chickens from different farms, we have chosen a combination of eight virulence-associated genes, which were, based on the results of previous research, considered good markers of APEC [5,7,9,10]. The genes *iroN* and *iutA* encode siderophore receptors and are associated with highly conserved virulence regions of ColV plasmids, as are the increased serum survival gene *iss* and outer membrane protease gene *ompT* [7]. The gene *cvaC* is a part of the operon of colicin V synthesis [11]. The *tsh* gene encodes for temperature-sensitive hemagglutinin, a ColV plasmid autotransporter presumably facilitating colonization of chicken tracheae [12]. The *frz* gene is a part of carbohydrate metabolic operon and probably contributes to stress adaptability of the strains with extraintestinal virulence [13]. The gene *felA* encodes for the F11 variant of P fimbriae [9]. Both these chromosomal genes are significantly associated with APEC strains from the B2 phylogenetic group [9,10].

Because of the diversity of APEC isolates, different vaccination strategies based only on serotype- or strain-specific immunity are not sufficiently effective in combating APEC infections in poultry farms [3,14]. Therefore, good zoohygienic conditions and adherence to biosecurity principles are needed to prevent clinical disease due to APEC in poultry farms. If disease does break out in the breeding stock, effective antibiotic treatment should be chosen to control it. However, the therapeutic use of antibiotics is complicated by the emergence of resistant populations of bacterial pathogens, not excluding APEC isolates. Antibiotic resistance is a global threat, and antibiotic use in livestock production is one source of it. Resistant animal pathogens can lead to treatment failure, which in turn causes economic losses to livestock producers, but they can also be a source of resistant bacteria/resistance genes that can pose a risk to human health [15]. To date, fluoroquinolones, especially enrofloxacin, have been used successfully to treat poultry colibacillosis, although enrofloxacin is not indicated for the treatment of coinfections in poultry, according to SPC information [16,17]. In addition, fluoroquinolones are classified as Category B “Restrict” according to the current classification of antibiotics by the European Medicines Agency, which precisely defines and severely restricts their use for animal treatment, and a complete ban on their use in veterinary medicine is even being considered to preserve their efficacy for human medicine [18].

Hatcheries are the main producer of chickens for commercial farms, which, in addition to the breeding farms themselves, can also be a potential source of pathogens and their new mutations colonizing one-day-old chicks. To test the possibility of transmission of pathogenic *E. coli* strains from hatcheries, the occurrence of APEC isolates in one-day-old broilers was monitored in samples collected immediately after transport from the hatchery to the farm.

## 2. Materials and Methods

### 2.1. Isolates

A total of 429 mixed swabs from 4290 one-day-old chicks (1 mixed swab was performed from 10 dead one-day-old chickens) were examined between August 2021 and July 2023 (24 months) during routine point-of-destination inspections at 12 poultry farms in the Czech Republic (Table 1).

The sources of the chicks were one hatchery in Germany, one hatchery in Hungary and one hatchery in the Czech Republic. Samples were taken randomly from different crates from the whole consignment, with 10 pieces from each supplier and from each group. Mixed samples from the 10 chicks in each group were collected using Transbak swabs containing Amies soil with activated charcoal (Dispolab s.r.o., Brno, Czech Republic). No animal care committee approval was necessary for the purposes of this study, as no animal work was required. The samples were collected from dead animals by practical veterinarians in cooperation with the farm owners and with their consent as part of the intake of one-day-old chickens to the farm. The authors declare that all applicable ethical guidelines were followed. Sampling was performed in all cases after sterile removal of the skin in the ventral part of the body after its disinfection with a swab moistened in ethyl alcohol (1 part ether to 1 part 65% alcohol) and after evaporation of the residual ethyl alcohol after 1 minute of exposure. Samples were taken from the abdominal wall around the umbilicus in all cases, as well as from the yolk sac and from organs of the body cavity (lungs, heart and liver). Cultures were performed on meat-peptone blood agar (MPBA) and Mac Conkey agar (both Lab Media Servis s.r.o., Jaromer, Czech Republic). Inoculated plates were incubated aerobically at 37 ± 1 °C for 18–24 h.

### 2.2. Identification of Isolates

Identification of *E. coli* isolates was performed on a MALDI-TOF MS (Bruker Daltonik GmbH, Bremen, Germany). All isolates identified as *E. coli* were screened via single or multiplex PCR reactions for the presence of genes encoding the virulence-associated factors *iroN*, *cvaC*, *iss*, *felA*, *iutA*, *frz* and *tsh* according to a recently reported methodology [19], which is based on methodologies described in previously published studies [5,6,7,10,11]. Briefly, the PCR reactions were performed with a Combi PPP Master Mix kit (Top-Bio, Vestec, Czech Republic) with the addition of 1 mM of each specific primer for individual Genes and 2 µL DNA from a lyzed bacterial suspension. The presence of the genes *iroN*, *cvaC* and *iss* was detected using a multiplex PCR under the following conditions: 94 °C for 3 min; 25 × 94 °C for 30 s, 58 °C for 30 s, 68 °C for 3 min; 72 °C for 10 min. The presence of the remaining genes was detected using single PCR under the following conditions:

*felA*: 94 °C for 3 min; 30 × 94 °C for 1 min, 61 °C for 1 min, 72 °C for 30 s; 72 °C for 10 min;
*iutA*: 94 °C for 3 min; 25 × 94 °C for 30 s, 57 °C for 30 s, 68 °C for 1.5 min; 72 °C for 10 min;
*frz*: 94 °C for 3 min; 26 × 94 °C for 1 min, 53 °C for 1 min, 72 °C for 1 min; 72 °C for 10 min;
*tsh*: 94 °C for 3 min; 26 × 94 °C for 1 min, 53 °C for 1 min, 72 °C for 1 min; 72 °C for 10 min.

The primers for targeting the virulence-associated genes (Table 2) were synthetized at Generi Biotech (Hradec Kralove, Czech Republic). Since the genes, primer sequences, reaction conditions and length of the products were taken from other methodologies, sequence analysis of the PCR products was not performed. If three or more of these genes were identified in an *E. coli* isolate, the isolate was designated as APEC.

### 2.3. Determination of Phenotypic Resistance of E. coli Isolates

All *E. coli* isolates were screened for resistance to six selected antibiotics (ampicillin, cefotaxime, tetracycline, doxycycline, enrofloxacin and florfenicol) and two antibiotic combinations (amoxicillin with clavulanic acid in a 2:1 ratio and trimethoprim with sulfamethoxazole in a 1:19 ratio) by determining the minimum inhibitory concentrations (MICs) using the microdilution method with kits manufactured at VÚVeL. Quality control of the MIC determination was carried out through concurrent testing of the reference strain *E. coli* ATCC 25922. Testing was performed in full accordance with internationally accepted Clinical Laboratory Standard Institute [20] and European Committee of Antimicrobial Susceptibility Testing (EUCAST) methodologies [21]. For each antibiotic, on the basis of the MICs determined, isolates were categorized into susceptibility categories—susceptible, intermediate (requiring an increased exposure time or antibiotic concentration for treatment) and resistant—using breakpoints. Clinical breakpoints for *E. coli* in relation to the individual production categories for either domestic chickens or poultry are not yet well defined; therefore, clinical breakpoints for the antimicrobial/pathogen combinations were derived from the human interpretation criteria [21] or animal interpretation criteria [22] to provide an indication of the distribution of isolates into susceptibility categories. The clinical breakpoints used are shown in Table 3. The MIC plates and confirmation methods were validated using reference strains of *Escherichia coli* (ATCC 25922) and *Staphylococcus aureus* (ATCC 25923).

### 2.4. Statistical Analysis

The data obtained were statistically analyzed using Statistica software (TIBCO Statistica^®^ 13.3.0). Descriptive analysis was performed at the significance level α = 0.05. The variability in the observations was expressed using a 95% confidence interval (IC). A binomial distribution was assumed for statistical comparison of differences in prevalence observations across farms. Two-tailed z-score tests were performed for the respective comparisons and the hypothesis H0: p1 = p2 = 0 was tested. The testing condition was *n* ≥ 30. A calculated p-value less than 0.05 (*p*-value < 0.05) was considered statistically significant.

## 3. Results

### 3.1. Prevalence of E. coli Isolates on Farms

*E. coli* isolates were found in the majority of samples from all farms (from 50% to 100%). *E. coli* isolates were obtained from 74.8 tested samples. The prevalence of *E. coli* isolates in collected samples is shown in Table 4. The two-sided test of the difference between two samples of relative frequencies showed a statistically significant difference in prevalence (%) on farm 9 from all other farms tested (1, 2, 4, 5 and 6, where *n* ≥ 30); for example, farm 1 vs. farm 9 had *p* = 0.0163. In general, for the tests comparing farms 1, 2, 4, 5 and 6, the calculated *p* > 0.05 was always the same.

The number of identified pathotypes based on virulence gene patterns on 12 farms is shown in Table 5. Pathotypes 2, 4 and 9 were identified most frequently, with pathotype 2 identified in 75 isolates (23.4% of all tested *E. coli* isolates).

### 3.2. Identification of Isolates and Pathotypes Associated with APEC

From 429 mixed samples, 321 *E. coli* strains (prevalence of 74.8%) were isolated, including 300 isolates defined as *E. coli* isolates associated with APEC via multiplex PCR. Based on the defined virulence genes, the isolates were classified into 31 pathotypes. According to the theory that three or more identified virulence genes classify *E. coli* isolates as APEC, 19 pathotypes of APEC isolates and 12 pathotypes of other *E. coli* isolates were found. No virulence gene was detected in 21 *E. coli* isolates (pathotype 6) (Table 6). 

**Figure 1:**
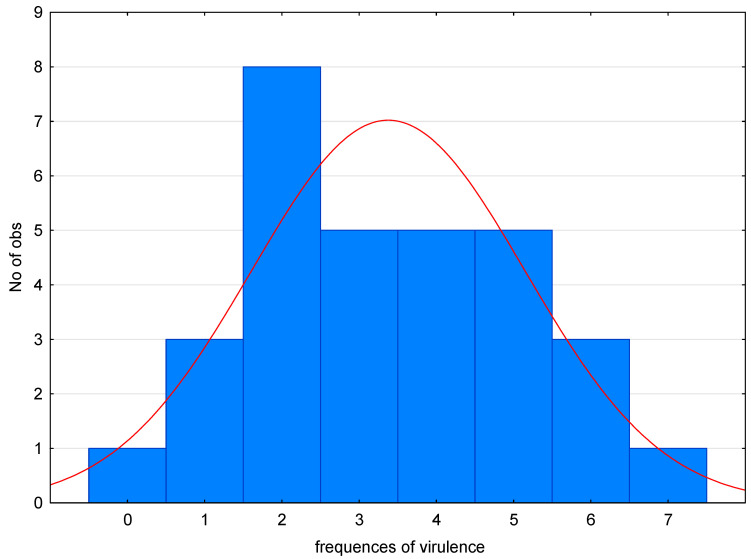
Prevalence of *E. coli* on farms.

Based on the number of detected virulence genes in individual pathotypes (frequencies of virulence), we performed a statistical evaluation of the frequency of findings of pathotypes with a certain number of APEC-associated genes (Figure 1). The X-axis is the number of APEC-associated genes and the Y-axis is the number of observations. From the graph, it can be inferred that each additional *E. coli* isolate found would likely be APEC (calculated probability: P = 0.613 ± 0.171), with three or more detected genes associated with APEC.

### 3.3. Phenotypic Resistance of E. coli Isolates

**Figure 2:**
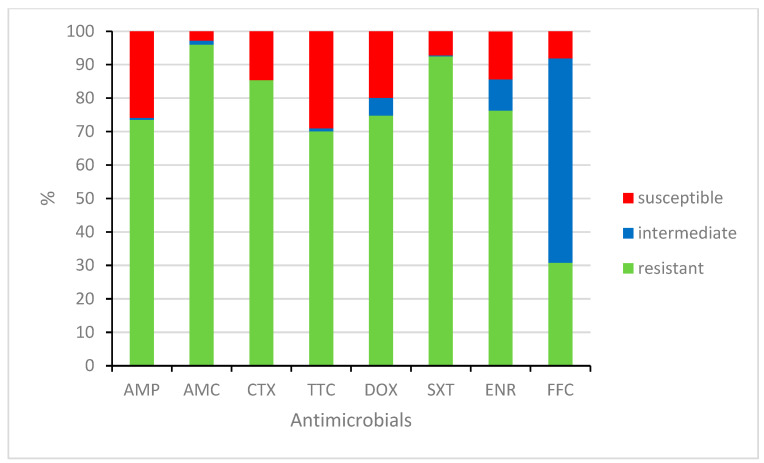
Susceptibilities/resistances of *E. coli* isolates (*n* = 321). AMP = ampicillin, AMC = amoxicillin/clavulanic acid, CTX = cefotaxime, TTC = tetracycline, DOX = doxycycline, SXT = trimethoprim/sulfamethoxazole, ENR = enrofloxacin, FFC = florfenicol.

Antibiotic susceptibilities/resistances were determined for all 321 *E. coli* isolates. The results are shown in Figure 2. The most susceptible isolates, which are expected to have the best efficacy against colibacillosis, were found to be susceptible to the combinations of antimicrobials amoxicillin/clavulanic acid (96.0%) and trimethoprim/sulfamethoxazole (92.5%). On the other hand, the tested isolates were most frequently resistant to tetracycline (29.0%), ampicillin (25.9%) and doxycycline (19.9%). Intermediate isolates are at risk of developing resistance to antibiotics. A high number of isolates (61.1%) were intermediate to florfenicol. A significant finding is that almost a quarter of the isolates were not susceptible to enrofloxacin, a representative of fluoroquinolones (9.3% intermediate isolates; 14.3% resistant isolates).

The phenotypic resistance profiles of the tested *E. coli* isolates are shown in Table 7. The resistance combinations found for each isolate are listed, with an indication of the number and percentage of isolates for each resistance profile. If an isolate was resistant to three or more of the tested antimicrobials or combinations of antimicrobials, it was designated as multidrug-resistant.

According to our findings, 15.9% of the tested isolates were susceptible to all tested antimicrobials and 35.5% of the isolates had no resistance, but these isolates were intermediate to some antibiotics. On the other hand, 20.5% of the isolates were identified as multidrug-resistant (8.7% of isolates were resistant to three antimicrobials; 7.3% to four antimicrobials; 3.6% to five antimicrobials; and 0.9% to six antimicrobials).

## 4. Discussion

Antibiotics are still a major tool in combating the occurrence or mitigating the course of infection in poultry colibacillosis [23,24]. However, the use of antibiotics for treatment also creates selection pressure for the emergence of resistance in the causative agents, which can lead to treatment failure and increased economic losses for farmers [24,25,26]. We found that only 15.9% of the isolates were susceptible to all tested antibiotics or combinations of antimicrobials. Resistance to antimicrobials that are contained in veterinary drugs (widely) used in livestock farms in the country warns of the risk of outbreaks of resistant strains. According to another study, *E. coli* strains isolated from birds are often resistant to more than one antibiotic, and the indiscriminate use of antibiotics is the most important factor in promoting the selection and spread of resistance [2,27,28].

The results of this study show a relatively significant difference in the number of resistant isolates between ampicillin (25.9%) and amoxicillin in combination with clavulanic acid (2.8%), an inhibitor of beta-lactamases, which are responsible for the emergence of resistance in penicillin antibiotics. According to the internationally accepted CLSI and EUCAST methodologies for testing bacterial susceptibility to antibiotics, the results are the same for ampicillin and amoxicillin. This clearly suggests that the combination of amoxicillin and clavulanic acid has the potential to increase the success rate of treatment compared with the use of amoxicillin or ampicillin alone. A relatively high percentage of isolates susceptible to cefotaxime, which is not registered for animal use. However, cefotaxime was tested for the purpose of detecting producers of broad-spectrum beta-lactamases (ESBLs) or AmpC-type beta-lactamases, which pose a high risk in terms of the spread of resistance in beta-lactams, was also found (85.4%) [20,21,22].

No resistance was detected in 165 isolates (51.4%), but the vast majority of these isolates (114; 35.5%) were intermediate to one or more antimicrobials, in most cases to florfenicol. Multidrug resistance (resistance to three or more antimicrobials) was detected in 67 isolates (20.5%). Some multidrug resistance profiles were consistent with those described in the literature in domestic chickens (*Salmonella* spp., *E. coli*), with possible transmission between genera/species of bacteria. For example, the *ogxAB* gene, which is responsible for reduced sensitivity or resistance to ciprofloxacin, was experimentally transferred from *Salmonella* spp. to *E. coli*. A phenotype of multidrug resistance to olaquindox, florfenicol, trimethoprim and tetracycline and reduced susceptibility to ciprofloxacin has also been demonstrated [29]. Another similar study described a similar phenotype of reduced susceptibility to olaquindox, tigecycline, nitrofurantoin and chloramphenicol, which facilitated the development of high levels of resistance to fluoroquinolones [30].

The development and spread of resistance in certain areas are closely related to national antibiotic policies and the use and consumption methods of particular antimicrobials in a given geographic area. Hence, the comparison of antimicrobial susceptibility testing results from different countries and geographic areas is controversial without knowing the other details of antimicrobial use. Nevertheless, it can be concluded that the results (very low percentage of *E. coli* isolates resistant to the combination of amoxicillin and clavulanic acid, and very high resistance to tetracyclines) published in this study do not differ much in principle from those in recently published papers. The exception is the results from testing potentiated sulfonamides with trimethoprim. Some publications describe a gradual decrease in the incidence of resistance in *E. coli* isolates from poultry [31], but other authors warn of more than 50% detection of *E. coli* isolates in poultry [32,33].

The question concerning the association of antimicrobial resistance with certain APEC virulence genotypes remains open. A typical feature of poultry *E. coli* is ColV plasmids, often carrying a specific profile of VAGs [7]. HGE (horizontal gene transfer) thus plays a key role in the genesis of the genetic diversity of APEC in terms of both virulence and resistance. High-risk “epidemic” clones of *E. coli*, described especially in humans, are often characterized by a combination of these two properties and, above all, an increased ability to colonize their host [34]. Similar to humans, several high-risk APEC clonal lines have recently been described in poultry, responsible for a non-negligible proportion of infections in commercial poultry. These are relatively phylogenetically distant and diverse lines, represented, in particular, by ST117, ST140/ST95, ST429 and ST23/ST88, which are characterized by specific genetic profiles with a certain degree of variability and also a varying tendency towards antimicrobial resistance [35]. Future monitoring and prevention of APEC infections could focus precisely on limiting the occurrence of these risk lines at different levels of the production chain [36].

The results of this study show a predominance of genotypes with 4-5 plasmid genes without (pathotypes 2 and 4) or in combination (pathotypes 5 and 9) with the chromosomal *frz* gene, indicating a generally very diverse poultry-associated *E. coli* population, in which virulent *E. coli* strains are dynamically generated through variation in mobile genetic elements on a suitable chromosomal background [37].

The relatively even distribution of these genotypes among different farms indicates their general occurrence, although a high number of isolates of a single profile on a single farm (as in the case of pathotype 2 with 21 isolates and pathotype 9 with 19 isolates on farm 2) may indicate an outbreak associated with a specific virulent clone [38,39]. In such cases, it would be advisable to proceed to sequencing the suspect isolates for the purpose of their closer characterization.

Treatment of *E. coli* infections usually requires the use of antimicrobial agents, the overuse of which often leads to the emergence and spread of new bacterial populations resistant to these agents, further complicating the situation. Predisposing factors for outbreaks of infectious diseases, including colibacillosis, in poultry farms are violations of welfare, animal nutrition and animal hygiene rules. This is due to rising energy and raw material prices and the need for farmers to generate ever higher profits.

The emergence of resistant strains of *E. coli* in poultry can also be prevented by reducing the use of antimicrobials and replacing them with alternative treatments and prevention methods such as plant extracts, immunopeptides, bacteriophages and, above all, high-quality autogenous vaccines. If antimicrobials are to be used, beta-lactam antibiotics should be preferred to other antibiotics, especially quinolones. However, beta-lactams (amoxicillin with clavulanic acid) are more expensive than quinolones and are therefore often preferred by breeders and veterinarians. In addition, clavulanic acid amoxicillin is not registered for poultry in many countries. Other antimicrobials such as tetracyclines, thiamulines or potentiated sulphonamides have longer withdrawal periods and are sometimes less well accepted by poultry.

## 5. Conclusions

Bacteriological testing of our samples confirmed that chickens as young as one day old can carry avian pathogenic *E. coli* (APEC) strains that can subsequently cause severe disease in both breeding and commercial poultry flocks with negative economic impacts on the poultry farmer. At the same time, they can also be a source of virulence and antimicrobial resistance for other animal populations, humans or the environmental microbiome. Our work also shows that contamination of chickens with various pathogens, including APEC strains, can occur not only during egg formation in the ovary and/or oviduct of breeding hens, but also in the environment of parental breeding and subsequently hatcheries, which, if hygiene is not maintained, can be a site for mixing “cocktails” of different genetic variants of microbial pathogens and various multidrug-resistant bacterial strains. These can then penetrate through the shell of the hatching eggs to the chicken embryos, or be spread by direct contact with the chicks or by aerosol inhalation by newly hatched chicks.

Effective prevention could be bacteriological monitoring of parents and grandparents, two-stage sorting of hatching eggs immediately after laying and subsequently in the hatchery, strict adherence to zoohygiene and welfare rules in breeding and collection of hatching eggs from as few sources as possible for hatching in one hatchery on one day. The hatchery should be thoroughly cleaned and disinfected and the premises and equipment should be cleaned and disinfected. To this end, a system of regular and random veterinary inspections of hatcheries and rearing facilities should be established or improved.

## Figures and Tables

**Table 1:** The types of breeds and the numbers of obtained *E. coli* isolates from individual farms during sampling from August 2021 to July 2023.

Farm No.	Kept Breed	No. of Collection Samples
1	ROSS 308 *	32
2	COBB 500, ROSS 308 †	84
3	COBB 500 *	19
4	ROSS 308 †	34
5	COBB 500 *	24
6	COBB 500 *	39
7	ROSS 308 *	27
8	ROSS 308 *	11
9	ROSS 308 *	29
10	Lohmann Brown ¤	10
11	ROSS 308 †	4
12	COBB 500, COBB 309 *	8

* Reproduction breeding for meat. † Broilers. ¤ Commercial laying hens.

**Table 2:** Primers used in PCR reactions.

Primer	Sequence	Gene	Product (bp)	Reference
iroN/F	ATC CTC TGG TCG CTA ACT G	*iroN*	847	[5]
iroN/R	CTG CAC TGG AAG AAC TGT TCT			
iss/F	ATC ACA TAG GAT TCT GCC G	*iss*	309	[5]
iss/R	CAG CGG AGT ATA GAT GCC A			
cvaC/F	CAC ACA CAA ACG GGA GCT GTT	*cvaC*	679	[11]
cvaC/R	CTT CCC GCA GCA TAG TTC CAT			
tsh/F	GGT GGT GCA CTG GAG TGG	*tsh*	620	[9]
tsh/R	AGT CCA GCG TGA TAG TGG			
iutA/F	GGC TGG ACA TCA TGG GAA CTG G	*iutA*	300	[7]
iutA/R	CGT CGG GAA CGG GTA GAA TCG			
frz/F	TCA GTA AGA ACG AAA GTG TG	*frz_orf4_*	565	[11]
frz/R	ACA GGA ACA ATC CCG TGG AT			
felA/F	GGT CAA SCA GCT AAA AAC GGT AAG G	*felA*	239	[9]
felA/R	CCT TCA GAA ACA GTA CCG CAA TTC G			

**Table 3:** Interpretation criteria used for susceptibility examination of APEC isolates in accordance with CLSI 2020 and EUCAST 2023 (concentrations are in mg/L).

Antimicrobials	Abbreviation	≤S ^1^	I ^2^	≥R ^3^	Source
Ampicillin	AMP	8	16	32	CLSI 2020
Amoxicillin/clavulanic acid	AMC	8/4	16/8	32/16	CLSI 2020
Cefotaxime	CEF	1	2	4	EUCAST
Tetracycline	TET	4	8	16	CLSI 2020
Doxycycline	DOX	4	8	16	CLSI 2020
Enrofloxacin	ENR	0.25	0.5–1	2	CLSI 2020
Florfenicol	FFC	4	8	16	CLSI 2020
Trimethoprim/sulfamethoxazole	SXT	2/38	-	4/76	CLSI 2020

^1^ Susceptible; ^2^ intermediate; ^3^ resistant. CLSI = Clinical Laboratory Standard Institute; EUCAST = European Committee on Antimicrobial Susceptibility Testing.

**Table 4:** Prevalence of *E. coli* isolates in collected samples.

Farm No.	No. of *E. coli* Isolates/No. of Collected Samples	Prevalence (%)
1	32/42	76.2
2	84/114	73.7
3	19/24	79.2
4	34/47	72.3
5	24/33	72.7
6	39/53	73.6
7	27/28	96.4
8	11/18	61.1
9	29/31	93.5
10	10/19	52.6
11	4/4	100
12	8/16	50
Total	321/429	74.8

**Table 5:** Number of all *E. coli* isolates and percentage of *E. coli* isolates associated with APEC originating from individual farms.

Pathotype	Farm											
	1	2	3	4	5	6	7	8	9	10	11	12
1	1						1		3			
2 ^1^	12	21	3	1	6	11	10	4	6	1		
3	2	1		7	4	3		1	2			
4	1	5	8	1	1	4		2	6		1	
5	1	6	3	1	2	2	2		2	5		
6	1	9		5	1	4				1		
7	2	2	1									
8		1					3					
9	2	19	1	1	2	2	1	1				3
10		2		4	3	2	3		5			2
11		1	1	2	1	3	1				3	2
12		1		1		2						
13	2	2			2			1	1			
14				1								
15	1					1	3		1			
16		1	1	2	1	1	1	1	1			
17	2			1								
18	2											
19	2					2		1				
20	1	1										
21		3		3								
22		5										
23		4		2	1	2			1	2		
24			1									
25				1								
26				1								
27							1					
28							1					
29									1			
30										1		
31												1
Total APEC isolate	31	75	19	29	23	35	27	11	29	9	4	8
% APEC Positive	96.9	89.3	100	85.3	95.8	89.7	100	100	100	90	100	100

^1^ Pathotypes with gene patterns associated with APEC are marked yellow.

**Table 6:** The list of *E. coli* pathotypes isolated from one-day-old chickens in period from August 2021 to July 2023.

Pathotype	*iroN*	*cvaC*	*iss*	*felA*	*iutA*	*frz*	*tsh*
1 ^1^	−	−	+	−	+	−	−
2 ^2^	+	+	+	−	+	−	+
3	−	−	−	−	+	+	−
4	+	+	+	−	+	−	−
5	+	+	+	−	+	+	−
6	−	−	−	−	−	−	−
7	+	−	+	−	+	+	+
8	+	+	+	−	−	+	−
9	+	+	+	−	+	+	+
10	+	−	+	−	−	−	−
11	+	+	+	−	−	−	−
12	−	−	−	−	+	−	+
13	+	−	+	−	−	+	−
14	−	−	−	−	+	+	+
15	+	−	+	−	+	−	+
16	+	−	+	−	+	−	−
17	+	+	−	−	−	−	−
18	−	+	−	−	+	−	−
19	+	+	+	−	−	−	+
20	+	+	+	+	+	+	+
21	+	−	−	−	−	−	−
22	+	−	−	−	+	−	−
23	−	−	−	−	+	−	−
24	+	+	+	−	−	+	+
25	+	+	+	+	−	+	−
26	+	+	+	+	+	+	−
27	−	−	+	−	−	−	−
28	−	−	+	−	+	+	+
29	+	−	+	+	+	+	+
30	+	−	−	−	−	+	−
31	−	−	+	−	+	+	−

^1^ Pathotypes with patterns of genes associated with APEC are marked in yellow. ^2^ Other pathotypes are marked in green.

**Table 7:** Resistance profiles of *E. coli* isolates (*n* = 321).

Profile	No. of Isolates	% of Tested Isolates
No resistance	51	15.9
Isolates with susceptible and intermediate AST results	114	35.5
AMP	17	5.3
CTX	8	2.5
FFC	11	3.4
TTC	5	1.6
ENR	3	0.9
SXT	1	0.3
AMP, AMC	1	0.3
AMP, CTX	1	0.3
AMP, ENR	4	1.2
AMP, FFC	3	0.9
AMP, SXT	2	0.6
AMP, TTC	5	1.6
CTX, ENR	3	0.9
CTX, TTC	6	1.9
DOX, FFC	1	0.3
ENR, FFC	1	0.3
TTC, DOX	16	5.0
TTC, ENR	1	0.3
AMP, AMC, TTC	1	0.3
AMP, AMC, CTX	1	0.3
AMP, AMC, ENR	1	0.3
AMP, AMC, SXT	2	0.6
AMP, CTX, TTC	5	1.6
AMP, SXT, ENR	1	0.3
AMP, TTC, SXT	2	0.6
AMP, TTC, DOX	5	1.6
CTX, TTC, DOX	5	1.6
SXT, ENR, FFC	1	0.3
TTC, DOX, ENR	4	1.2
AMP, AMC, SXT, ENR	1	0.3
AMP, CTX, TTC, DOX	2	0.6
AMP, CTX, TTC, ENR	1	0.3
AMP, CTX, TTC, SXT	2	0.6
AMP, TTC, DOX, ENR	9	2.8
AMP, TTC, DOX, FFC	2	0.6
AMP, TTC, DOX, SXT	1	0.3
AMP, TTC, SXT, ENR	1	0.3
CTX, TTC, DOX, ENR	2	0.6
CTX, TTC, DOX, FFC	1	0.3
TTC, DOX, SXT, ENR	1	0.3
TTC, DOX, SXT, FFC	1	0.3
AMP, AMC, TTC, DOX, ENR	1	0.3
AMP, CTX, TTC, DOX, ENR	4	1.2
AMP, CTX, TTC, DOX, FFC	2	0.6
AMP, CTX, TTC, SXT, FFC	1	0.3
AMP, TTC, DOX, SXT, ENR	2	0.6
TTC, DOX, SXT, ENR, FFC	2	0.6
AMP, CTX, TTC, DOX, SXT, ENR	2	0.6
AMP, AMC, CTX, TTC, DOX, ENR	1	0.3
Total of tested strains	321	100

AST = antimicrobial susceptibility testing; AMP = ampicillin; AMC = amoxicillin/clavulanic acid; CTX = cefotaxime; TTC = tetracycline; DOX = doxycycline; SXT = trimethoprim/sulfamethoxazole; ENR = enrofloxacin; FFC = florfenicol.

## Data Availability

Raw data supporting the conclusions of this study are available from the authors upon request.
